# Lard—Its Adulteration, Etc.

**Published:** 1889-05

**Authors:** 


### LARD—ITS ADULTERATION WITH COTTON—SEED OIL AND DETECTION THEREOF.

The purity of lard for use as an ointment basis is a matter of some importance, and consequently the British Pharmacopoeia directs it to be made from the perfectly fresh internal fat of the abdomen of the hog, and according to the to the same authority, it should respond to the following characters and tests: "A soft white fatty substance, melting at 100 degrees F. (37.8 C). Has no rancid odor, dissolves entirely in ether, distilled water in which it has been boiled when cooled and filtered gives no precipitate with nitrate of silver, and is not rendered blue by the addition of solution of iodine." These tests are very good so far as they go, but they are, unfortunately, not sufficient for the misapplied ingenuity of the day.

For several weeks past I have tried these and other tests with the object of finding the most reliable and expeditious, and my experience is that those dependent upon the deduction of silver nitrate are the best, and the following *modus operandi*, has given me results that are entirely satisfactory and reliable, and only requires a few minutes time:

1. Make a test solution containing 5 parts of silver nitrate and one part of nitric acid (sp. gr. 1 42) in 100 parts of rectified spirit (sp. gr. 838).

2. Melt a small quantity of the lard to be tested in a water-bath and pour about 100 grains of it into a dry test-tube about one-half inch in diameter. To this add 20 grain measures of the above mentioned test solution and place the tube in boiling water for five minutes, taking care that no water enters it.

Pure lard remains perfectly white, but if adulterated with cotton-seed oil it assumes a more or less olive-brown color, according to the amount present. The color is best seen when the lard sets, and it saves time to put the test tube direct from the boiling into a vessel of cold water.

The presence of 5 per cent. of cotton seed oil in lard gives a very decided olive-brown coloration with this test, and 1 per cent. gives a color quite distinct from genuine lard. The addition of nitric acid to the test solution is intended to neutralize any trace of alkali or alkaline carbonate that may be present, and it also prevents a slight reduction of the silver nitrate which takes place in genuine lard. It must not be forgotten that some samples of lard might possibly contain sodium chloride, though I have never met with any, in which case the silver nitrate would be precipitated as chloride instead of reacting on the oil, but this would be at once seen by the white curdy precipitate that would be formed.—*Anti Adulteration Journal.*

